# Some Remarks

**Published:** 1891-09

**Authors:** 


### SOME REMARKS.

THE voice of the medical reformer is now heard in the land. And it is the rights and freedom of that worthy class of American citizens, the doctor, that he wishes principally to modify or abridge. His usual habitat is in the legislature. Here in Georgia he proposes to make it a misdemeanor for physicians to get drunk. During the present session he nursed for a few days a measure over in the Capitol, requiring all Georgia Medical Colleges to inaugurate a three-year course. This infant, which deserved to live, smiled faintly at first, then gasped and died.

Out in Missouri also he is vexing his massive brain with the three-year course; is also struggling with the ever-present and ever-interesting matter of *prostitution*; and lastly, but not least, he proposes to limit physicians' fees to one dollar per visit, and fifty cents for office consultation.

In New York, not very long ago, the legislature made an enactment, which, putting it very mildly, has not been altogether an unmixed blessing. Now they propose to remedy all difficulties by giving the licensing power to the faculties of certain medical colleges. Imagine the passage of such a measure in New York! Compared with the confusion which would result, the alleged Babel incident would be a concord of sweet sounds, and Pandemonium a Quaker meeting.

SPEAKING of medical legislation, cultured and cultivated Massachusetts deserves the warm congratulations of all lovers of medical progress and reform. The comedians of her General Assembly have passed a bill—called a *law*, through courtesy—which requires simply and only a *register* to be kept *of all persons who desire to practice medicine in the State.* Really, New England must be coming to the front, and we suspect that ere long enlightened and hyperæsthetic Massachusetts will be in line with our esteemed and phlegmatic sister, Alabama.

We suggest to the funny legislators of the classic Bay State the following motto for their little bill, and pray the shades of Horace to excuse our liberties with his Latin : *Parturibant montes; nascitur ridiculus mus.*

THE latest sensation in medical and lay circles abroad seems to be the so-called *scandal* which has been considerably exercising the nervous Parisian mind of late.

It seems that as far back as four years ago, an enterprising surgeon (whose name is withheld for prudential reasons), imbued with an ambitious spirit of experiment, inoculated the healthy mamma of a female patient with a small portion of a sarcoma removed from the other breast. The inoculation "took," so to speak, and in two months a hard nodule, the size of an almond, developed. Sections of both tumors were submitted to Professor Cornil who pronounced them identical and composed of fasciculated sarcomatous tissue. Sometime after the second tumor was removed the patient died from some intercurrent condition, and the autopsy failed to show the existence of sarcoma in any other part of the body.

An analogous experiment was made on another patient with a cylindrical epithelioma, but the structure of the secondary tumor was not ascertained, as the patient refused to allow its removal.

These results were reported by Professor Cornil to the French Academy of Medicine in June. The *N. Y. Medical Journal*

remarks: "These experiments are obviously, when viewed from a scientific standpoint, of vital importance, and, considered in connection with recent researches on the parasitic nature of malignant growths, cannot fail to be of great use to future investigators. But morally no possible excuse can be found for such trifling with a precious human life, and the conduct of the anonymous surgeon in question fully merits the condemnation passed on it by M. Le Fort and M. Moutard-Martin, whose remarks were cheered to the echo by the members of the *Academie* present on the occasion. It is only just to add that Professor Cornil hastened to associate himself with the sentiments expressed by his colleagues, alleging that his sole motive in bringing the matter to the notice of his fellow academicians was a purely scientific one. The lay press was, of course, not behindhand in indignantly protesting against such an inhuman proceeding. No one who has the privilege of knowing Professor Cornil, who is kindness itelf, would for one instant entertain the belief that this distinguished pathologist had been actuated by aught but the most elevated humanitarian motives in making public—after a lapse of four years, be it said—the results of an experiment whose practical importance cannot well be exaggerated, however severely one may condemn the proceedings of its author."

---

Dr. Mark O'Daniel, so long associated with the State Insane Asylum, has resigned his position, in order to resume general practice. He is now renewing his medical studies in New York, and upon his return will locate in Macon. As far as possible he wishes to make a specialty of nervous and mental diseases. Dr. W. A. O'Daniel, formerly of Macon, and Dr. Patterson have been elected on the house staff at Asylum.

